# Multiscale reaction-diffusion simulations with Smoldyn

**DOI:** 10.1093/bioinformatics/btv149

**Published:** 2015-03-18

**Authors:** Martin Robinson, Steven S. Andrews, Radek Erban

**Affiliations:** ^1^Mathematical Institute, University of Oxford, Radcliffe Observatory Quarter, Woodstock Road, Oxford, OX2 6GG, United Kingdom and; ^2^Fred Hutchinson Cancer Research Center, 1100 Fairview Ave N, Seattle, WA 98109, United States

## Abstract

**Summary:** Smoldyn is a software package for stochastic modelling of spatial biochemical networks and intracellular systems. It was originally developed with an accurate off-lattice particle-based model at its core. This has recently been enhanced with the addition of a computationally efficient on-lattice model, which can be run stand-alone or coupled together for multiscale simulations using both models in regions where they are most required, increasing the applicability of Smoldyn to larger molecule numbers and spatial domains. Simulations can switch between models with only small additions to their configuration file, enabling users with existing Smoldyn configuration files to run the new on-lattice model with any reaction, species or surface descriptions they might already have.

**Availability and Implementation:** Source code and binaries freely available for download at www.smoldyn.org, implemented in C/C++ and supported on Linux, Mac OSX and MS Windows.

**Contact:** martin.robinson@maths.ox.ac.uk

**Supplementary Information**: Supplementary data are available at *Bioinformatics* online and include additional details on model specification and modelling of surfaces, as well as the Smoldyn configuration file used to generate Figure 1.

## 1 Introduction

In recent years, computational simulations for modelling spatial biochemical networks and intracellular systems have grown in popularity. The rapid increase in computation power and software complexity have increased the range of applicability of these tools to larger and more complex biological systems, while including important stochastic, geometrical (boundary) and multiscale temporal and spatial effects. Computational simulations have been successfully used to understand cellular systems such as cell division in *Escherichia coli* (Fange and Elf, 2006), spatio-temporal correlations in the Mitogen-Activated Protein Kinase (MAPK) pathway (Takahashi *et al.*, 2010) and actin dynamics in filopodia (Zhuravlev and Papoian, 2009).

Smoldyn (www.smoldyn.org) is a spatial stochastic simulator that uses an accurate off-lattice method to simulate user-defined reaction-diffusion networks on arbitrary domains and surfaces. It uses a readable, plain-text configuration file to specify molecular species, reactions, simulation surfaces or compartments and has a run-time command feature that allows users to specify either observations (e.g. concentration profiles, total molecule numbers) or manipulations of the system (e.g. moving surfaces, changing reaction or diffusion rates).

This article presents a major update of the Smoldyn package, which incorporates a new on-lattice model and its coupling with the existing off-lattice model. A significant new feature is the ability to setup multiscale simulations that can use the accurate off-lattice models in specific regions of interest, coupled with a coarse but computationally efficient on-lattice model for the rest of the domain (Erban *et al.*, 2013).

**Fig. 1.**
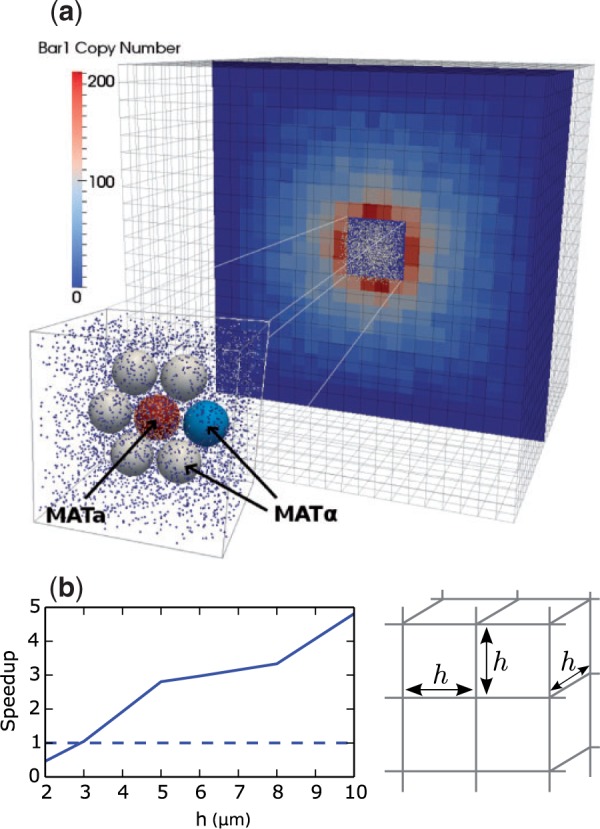
(**a**) Multiscale Smoldyn simulation of yeast signalling between haploid Saccharomyces cerevisiae of opposite mating types (MATa and MATα; Andrews *et al.*, 2010). The centre region contains a detailed off-lattice model of several MATa and MATα cells, which is coupled to a coarse on-lattice model to describe the diffusion and interaction of a pheromone (α-factor) and a pheromone-degrading protease (Bar1) within the surrounding environment. (**b**) Speed-up versus lattice spacing *h* that can be achieved by using the on-lattice model for the bulk of the domain (speed-up equals 1 for a purely off-lattice simulation). The simulation domain is a 100 × 100 × 100 μm cube with an absorbing boundary. The gradient of bound α-factor across the central cell (a measure of simulation accuracy) did not vary with *h* over the range considered, and the mean was within 1% of that obtained from the purely off-lattice simulation

Multiscale simulations of this nature are useful for modelling unbounded or large domains where a large portion of the domain is homogeneous or has limited concentration gradients. An example is illustrated in Figure 1, which shows a multiscale model of yeast signalling using Smoldyn as well as the speed-up achieved versus lattice resolution *h*. Using a coarse on-lattice model allows the simulation of a large domain with minimal computational effort. It is also flexible enough to allow for the addition of surfaces and/or other off-lattice subdomains.

Similar software packages that couple off-lattice and on-lattice methods have been described by Hellander *et* *al.* (2012; URDME), Klann *et al.* (2012) and VCell (Slepchenko *et al.*, 2003), which supports overlapping space multiscale simulation for deterministic and stochastic off-lattice models. One advantage of multiscale modelling in Smoldyn is its accuracy, as discussed in Section 5. Another significant advantage is Smoldyn’s flexibility and large feature-set for specifying an increasingly wide variety of spatial models. It is primarily a tool for application-focused users, and features comprehensive documentation and example configurations scripts. Smoldyn incorporates a surface description language, where complicated geometries (e.g. cellular membranes) can be built up from individual elements. Molecular species can reflect, be absorbed or transmit through the surfaces, or associate/disassociate and diffuse along the front or back of each surface, reacting with other molecules in solution or bound to the surface. The surface description language, as well as reflection, absorption and transmitting actions have all been incorporated into the new on-lattice model so surfaces can extend across both on-lattice and off-lattice model subdomains (Supplementary Information).

## 2 Methods

The off-lattice model, that has been at the heart of Smoldyn, treats each molecule as a point in a continuous spatial domain. These molecules diffuse in solution or along surfaces, interact with surfaces and undergo zeroth, first and second order reactions, according to a constant timestep algorithm based on the Smoluchowski model (Andrews and Bray, 2004; Andrews, 2012). The new on-lattice model uses a regular array of connected lattice sites. Molecules can diffuse by jumping between neighbouring lattice sites and react with molecules at the same site. The model can be mathematically described as a discrete space continuous time Markov chain and is implemented using the Next Subvolume Method (NSM) (Elf and Ehrenberg, 2004).

The coupling between the on-lattice and off-lattice regions uses the Two Regime Method (TRM) (Flegg *et al.*, 2012; Flegg *et al.*, 2014), which correctly calculates the diffusion flux of species through an interface between the subdomains. Robinson *et al*. (2014) provides an error analysis and generalisation of the TRM to moving interfaces (ATRM).

The on-lattice model is ideally used in regions of high molecule number and/or relatively homogeneous molecule distributions. The latter condition is also helpful to reduce the diffusion error due to the coupling (TRM), which vanishes as the net flux of molecules over the interface goes to 0. If this is not possible, then the minimum coupling error is achieved by using a balance between the lattice spacing *h* and off-lattice timestep Δt so that $h\approx \sqrt{\pi D_{S}\Delta t}$, where *D_S_* is the diffusion constant for species *S*. Away from the interface, the spatial discretization introduced by the lattice means that the diffusion error is slightly increased with order $O(h^{2})$, particularly near surfaces, so in regions, where high spatial accuracy or complex geometry is required the off-lattice model is preferred. The performance of the coupling algorithm between the models scales with the flux of molecules across the interface, meaning that the performance is generally limited by the off-lattice and on-lattice models individually, rather than the coupling.

## 3 Conclusion

The addition of an efficient on-lattice model to Smoldyn and the ability to setup multiscale simulations allows the simulation of larger domains and molecule numbers, while still allowing the use of an off-lattice model for accurate treatment of surface geometries, surface-bound species and high concentration gradients. This is advantageous for the simulation of highly heterogeneous reaction-diffusion processes, e.g. the growth of actin filaments (Erban *et al.*, 2013) or intracellular calcium signalling (Flegg *et al.*, 2013).

## Supplementary Material

Supplementary Data
