# Supplementary material for: Multiscale reaction-diffusion simulations with Smoldyn
Source: Bioinformatics. 2015 Mar 18;31(14):2406–8. doi: 10.1093/bioinformatics/btv149 (PMC4495299; doi:10.1093/bioinformatics/btv149)
Supplement: Supplementary Data [file supp_31_14_2406__index.html]

Multiscale Reaction-Diffusion Simulations with Smoldyn — Multiscale reaction-diffusion simulations with Smoldyn — Multiscale reaction-diffusion simulations with Smoldyn — Supplementary Data 

# Multiscale reaction-diffusion simulations with Smoldyn

## Supplementary Data

files

**Files in this Data Supplement:**

- Supplementary Data - pdf file
- Supplementary Data - pdf file
