# Supplementary material for: Multiscale reaction-diffusion simulations with Smoldyn
Source: Bioinformatics. 2015 Mar 18;31(14):2406–8. doi: 10.1093/bioinformatics/btv149 (PMC4495299; doi:10.1093/bioinformatics/btv149)
Supplement: Supplementary Data [file supp_btv149_ModelSpecificationAndSurfaces.pdf]

1 Model Specification

Incorporating the on-lattice model into existing Smoldyn simulations can be done with only small changes to the configuration file, allowing users to effectively re-use existing Smoldyn models.

To construct a multiscale simulation, the user first defines a port surface to connect the two models. For example, the configuration file extract shown in Listing 1 creates a rectangle port surface normal to the x-axis at $x = 50$. This port will allow molecules to cross from the off-lattice region to the lattice region, and vice versa. Of course, it is also possible to setup purely off-lattice or on-lattice simulation, in which case no ports are needed.

Listing 1: specify port surface

```
start_surface port_surface
action front all port
panel rect -0 50 0 0 100 100
end_surface

start_port my_port
surface port_surface
face front
end_port
```

Listing 2: setup on-lattice model

```
start_lattice my_lattice
type nsv
port my_port
boundaries 0 47 100
boundaries 1 0 100 p
boundaries 2 0 100 p
lengthscale 3 3 3
species all
reactions all
surfaces all
end_lattice
```

Listing 2 shows how to create the lattice domain and setup the parameters, including which species, reactions and surfaces to include. These items are assumed to be previously specified using the standard Smoldyn input file format. The listing shown here creates a lattice (periodic in the x and y axis directions) over the domain $(47, 0, 0) \leq (x, y, z) < (100, 100, 100)$ with an isotropic lattice spacing $h = 3$.

In this case, Smoldyn will attempt to add all the species, reactions and surfaces previously defined to the on-lattice model. All reactions that occur in solution are supported by the on-lattice model. All surface actions that occur across the surface (i.e. reflection, absorption, transmission) are also supported. Surface-bound species that diffuse and react along the surface geometry are only supported by the off-lattice model.

2 Output

The user can generate output data using Smoldyn's command system, or see the current state of the simulation in Smoldyn's OpenGL visualisation window. Alternatively, the full state of the molecules can be written out using the standard VTK file format (vtk.org) for later post-processing and visualisation. For example, all the rendered figures in this paper were generated from VTK files using Paraview (www.paraview.org).

3 Surfaces

In the off-lattice model, surfaces are defined by specifying panel shapes (rectangles, triangles, discs, hemispheres, spheres and cylinders), which can be combined to form more complicated geometries (see Figure 1a). These same surface descriptions can be supplied to the on-lattice model, which effectively discritizes the surfaces to be along lattice boundaries, based upon the middle of the lattice site (see Figures 1b & 1c). The on-lattice model implements surface actions (reflection, absorption, transmission) by modifying the diffusion jump rates between lattice sites on either side of the surface.

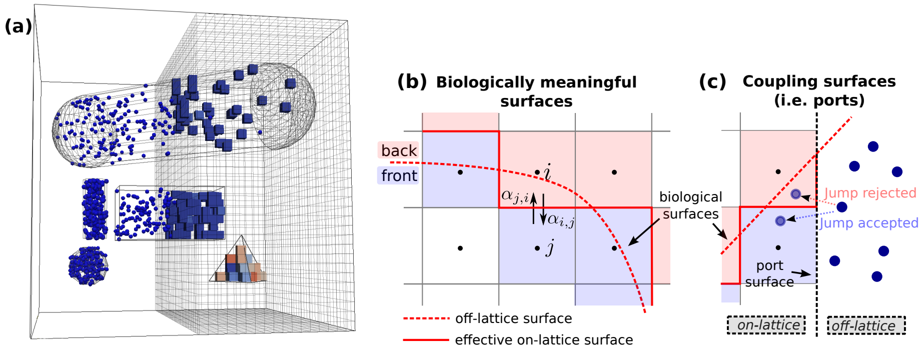

Figure 1: **(a)** Visualisation illustrating different (reflective) surface geometries in a hybrid simulation. **(b)** Surfaces modify the diffusion jump rates $\alpha_{i,j} = \alpha_{j,i} = \alpha_S$ for species S from lattice sites i to j, where i and j are on opposite sides of the surface. For example, a reflecting or absorbing boundary condition on the front of the surface would both set $\alpha_{i,j} = 0$, with the absorbing condition also adding a $S \to \emptyset$ reaction to j, with reaction rate $k = \alpha_S$. **(c)** In the off-lattice regions, surfaces are curved and off-lattice, but in the on-lattice regions they are discritized according to the lattice resolution. Therefore, it is possible for molecules to attempt jumps from the off-lattice to on-lattice regions that would be allowed under the former model but not the latter. Smoldyn checks these conditions and ensures accurate diffusion jumps that are consistent with each model within its own domain.
