# Supplementary material for: Multiscale reaction-diffusion simulations with Smoldyn
Source: Bioinformatics. 2015 Mar 18;31(14):2406–8. doi: 10.1093/bioinformatics/btv149 (PMC4495299; doi:10.1093/bioinformatics/btv149)
Supplement: Supplementary Data [file supp_btv149_yeastSignalling.pdf]

1 Configuration File

Reproduced below is the configuration file for the multiscale yeast signalling example shown in Fig. 1 of the main paper. This file is derived from the yeast signalling Smoldyn simulation first published in Andrews *et al.* (2010).

The configuration file takes in four parameters: WITHLATTICE, SPACING, INNER and TWOINNER.

WITHLATTICE define this parameter to enable a multiscale lattice simulation. If undefined a purely off-lattice simulation is used.

SPACING sets the lattice spacing (h in the main paper)

INNER sets the side length of the inner off-lattice region. This must be a multiple of SPACING

TWOINNER set this parameter to two times INNER

For example, the following runs the simulation using $h = 2$ and with the off-lattice region side length of 10

```
smoldyn Bar29Paramscan.txt --define WITHLATTICE=1 --define SPACING=2 --define
      INNER=10 --define TWOINNER=20 -tq
```

The full configuration file is listed below:

```
# Multiscale Bar1 Smoldyn simulation
# by Steve Andrews and Martin Robinson, 19/12/2014
# Ref: Andrews, Addy, Brent, Arkin, "Detailed simulations of cell biology
    with Smoldyn 2.1", PLoS Comp. Biol. 2010
# This file is Bar29_Multiscale.txt, from Bar29.txt
# This is Bar1+, 1 target cell, no Bar1 adsorption
# Units: microns and seconds

define WITHBAR1

# *** output file ***
define  OUTFILE1        FILEROOTout_SPACING.txt
define  OUTFILE2        FILEROOTout2_SPACING.txt
define  OUTFILE3        vtkout_SPACING/FILEROOT


# *** time ***
define TIMEEND 500

# *** boundaries ***
define  XLO     -10
define  XHI     10
define  YLO     -10
define  YHI     10
define  ZLO     -10
define  ZHI     10
define OUTER 50
define TWOOUTER 100
define LATTICE 60


# *** model parameters ***
define  NGPCR   6622    # number of GPCR
define  K1t     250     # target alpha production rate
define  K1c     12.5    # challenger alpha production rate
```

```
define  K4      100     # Bar1 production rate in um^-2/s
define  K5      5.15    # Bar1-alpha reaction rate, diff. limit is 10.3 um^3/
    s
define  K6      0.008303        # alpha binding to GPCR, *** 2x
define  K7      0.02    # alpha unbinding from GPCR, *** 2x

#graphics opengl
#graphic_iter 10000
#frame_thickness 0
#accuracy 10

dim 3
species Bar1 alpha GPCR GPCRalpha
max_mol 300000
boxsize 0.5

molecule_lists list3 list4 list1 list2 list5

mol_list alpha(fsoln) list1
mol_list alpha(up) list2
mol_list GPCR(up) list3
mol_list GPCRalpha(up) list4
mol_list Bar1(fsoln) listhidelinks5
mol_list Bar1(up) list2

difc Bar1(fsoln) 27
difc alpha(fsoln) 132

color Bar1(fsoln) 0 1 0
color Bar1(front) 0 1 0
color alpha(fsoln) 0 0 0
color GPCR(up) 0 0 1
color GPCRalpha(up) 1 0 0.2

display_size alpha(fsoln) 1
display_size alpha(up) 0
display_size Bar1(all) 1
display_size GPCR(up) 2
display_size GPCRalpha(up) 2

time_start 0
time_stop TIMEEND
time_step 0.02

boundaries 0 XLO XHI
boundaries 1 YLO YHI
boundaries 2 ZLO ZHI

max_surface 5

#start_surface sides
#polygon both none
#unbounded_emitter front Bar1 K4 0 0 0
#unbounded_emitter front alpha K1t 5.5 0 0
#unbounded_emitter front alpha K1c 2.75 4.7632 0
#unbounded_emitter front alpha K1c -2.75 4.7632 0
#unbounded_emitter front alpha K1c -5.5 0 0
#unbounded_emitter front alpha K1c -2.75 -4.7632 0
#unbounded_emitter front alpha K1c 2.75 -4.7632 0
#read_file ellipse_12_12.txt
#end_surface

start_surface cell
action both all reflect
polygon both face
color both 0.8 0.8 0.8
max_panels sphere 1
panel sphere 0 0 0 2.5 20 20
```

```
end_surface

start_surface alphatarget
polygon both face
color both 0.3 0.3 0.3
max_panels sphere 6
panel sphere 5.5        0       0 2.5 20 20
end_surface

start_surface alphachallenge
polygon both face
color both 0.5 0.5 0.5
max_panels sphere 6
panel sphere 2.75       4.7632  0 2.5 20 20
panel sphere -2.75      4.7632  0 2.5 20 20
panel sphere -5.5       0       0 2.5 20 20
panel sphere -2.75      -4.7632 0 2.5 20 20
panel sphere 2.75       -4.7632 0 2.5 20 20
end_surface

start_surface absorption_surface                # the port is a 20x20x20 box
    in the middle of the particle space
action both all absorb
color front 0.2 0 0 0.5
color back 0 0 0
polygon both face
panel rect +0 -OUTER -OUTER -OUTER TWOOUTER TWOOUTER
panel rect -0 OUTER -OUTER -OUTER TWOOUTER TWOOUTER
panel rect +1 -OUTER -OUTER -OUTER TWOOUTER TWOOUTER
panel rect -1 -OUTER OUTEhidelinksR -OUTER TWOOUTER TWOOUTER
panel rect +2 -OUTER -OUTER -OUTER TWOOUTER TWOOUTER
panel rect -2 -OUTER -OUTER OUTER TWOOUTER TWOOUTER
end_surface

surface_mol NGPCR GPCR(up) cell all all

reaction_surface alphatarget rxn1t 0 -> alpha(fsoln) K1t # alpha production
reaction_surface alphachallenge rxn1c 0 -> alpha(fsoln) K1c # alpha
    production

ifdefine WITHBAR1
  reaction_surface cell rxn4    0 -> Bar1(fsoln) K4 # Bar1 production
  reaction rxn5 Bar1(fsoln) + alpha(fsoln) -> Bar1(fsoln) K5 # Bar1 protease
      on alpha
endif

reaction rxn6 GPCR(up) + alpha(fsoln\usepackage) -> GPCRalpha(up) K6 # GPCR-
    alpha binding
reaction rxn7 GPCRalpha(up) -> GPCR(up) + alpha(fsoln) K7 # GPCR-alpha
    unbinding
product_placement rxn7 pgemmax 0.2

ifdefine WITHLATTICE
        start_surface portsurf       # the port is a 20x20x20 box in the
            middle of the particle space
        action front all port
        color front 0.2 0 0 0.5
        color back 0 0 0
        polygon both face
        panel rect +0 -INNER -INNER -INNER TWOINNER TWOINNER
        panel rect -0 INNER -INNER -INNER TWOINNER TWOINNER
        panel rect +1 -INNER -INNER -INNER TWOINNER TWOINNER
        panel rect -1 -INNER INNER -INNER TWOINNER TWOINNER
        panel rect +2 -INNER -INNER -INNER TWOINNER TWOINNER
        panel rect -2 -INNER -INNER INNER TWOINNER TWOINNER
        end_surface

        start_port myport
```

```
        surface portsurf
        face front
        end_port

        start_lattice outer_lattice
        type nsv
        port myport
        boundaries 0 -LATTICE LATTICE
        boundaries 1 -LATTICE LATTICE
        boundaries 2 -LATTICE LATTICE
        lengthscale SPACING SPACING SPACING
        species all
        surfaces absorption_surface
        ifdefine WITHBAR1
          reactions rxn5        # Bar1 protease on alpha
        endif
        end_lattice
endif

cmd @ 0 set reaction_rate rxn1t 15.625
cmd @ 500 set reaction_rate rxn1t 31.25
cmd @ 1000 set reaction_rate rxn1t 62.5
cmd @ 1500 set reaction_rate rxn1t 125
cmd @ 2000 set reaction_rate rxn1t 250
cmd @ 2500 set reaction_rate rxn1t 500
cmd @ 3000 set reaction_rate rxn1t 1000
cmd @ 3500 set reaction_rate rxn1t 2000
cmd @ 4000 set reaction_rate rxn1t 4000

cmd @ 0 set reaction_rate rxn1c 0.78125
cmd @ 500 set reaction_rate rxn1c 1.5625
cmd @ 1000 set reaction_rate rxn1c 3.125
cmd @ 1500 set reaction_rate rxn1c 6.25
cmd @ 2000 set reaction_rate rxn1c 12.5
cmd @ 2500 set reaction_rate rxn1c 25
cmd @ 3000 set reaction_rate rxn1c 50.0
cmd @ 3500 set reaction_rate rxn1c 100.0
cmd @ 4000 set reaction_rate rxn1c 200.0

output_files stdout OUTFILE1 OUTFILE2

cmd B molcountheader stdout
cmd B molcountheader OUTFILE2
cmd i 0 TIMEEND 2 writeVTK OUTFILE3
cmd i 0 TIMEEND 2 molcount stdout
cmd i 0 TIMEEND 2 molcount OUTFILE2
cmd i 0 TIMEEND 2 molmoments GPCRalpha(up) OUTFILE1

end_file
```

References

Andrews, S. S., Addy, N. J., Brent, R., and Arkin, A. P. (2010). Detailed simulations of cell biology with smoldyn 2.1. *PLoS Computational Biology*, **6**(3), e1000705.
